# Validation of a New Method for Testing Provider Clinical Quality in Rural Settings in Low- and Middle-Income Countries: The Observed Simulated Patient

**DOI:** 10.1371/journal.pone.0030196

**Published:** 2012-01-23

**Authors:** Tin Aung, Dominic Montagu, Karen Schlein, Thin Myat Khine, Willi McFarland

**Affiliations:** 1 Research Department, Population Services International, Yangon, Myanmar; 2 Department of Epidemiology, University of California San Francisco, San Francisco, California, United States of America; McGill University, Canada

## Abstract

**Background:**

Assessing the quality of care provided by individual health practitioners is critical to identifying possible risks to the health of the public. However, existing assessment methods can be inaccurate, expensive, or infeasible in many developing country settings, particularly in rural areas and especially for children. Following an assessment of the strengths and weaknesses of the existing methods for provider assessment, we developed a synthesis method combining components of direct observation, clinical vignettes, and medical mannequins which we have termed “Observed Simulated Patient” or OSP. An OSP assessment involves a trained actor playing the role of a ‘mother’, a life-size doll representing a 5-year old boy, and a trained observer. The provider being assessed was informed in advance of the role-playing, and told to conduct the diagnosis and treatment as he normally would while verbally describing the examinations.

**Methodology/Principal Findings:**

We tested the validity of OSP by conducting parallel scoring of medical providers in Myanmar, assessing the quality of their diagnosis and treatment of pediatric malaria, first by direct observation of true patients and second by OSP. Data were collected from 20 private independent medical practitioners in Mon and Kayin States, Myanmar between December 26, 2010 and January 12, 2011. All areas of assessment showed agreement between OSP and direct observation above 90% except for history taking related to past experience with malaria medicines. In this area, providers did not ask questions of the OSP to the same degree that they questioned real patients (agreement 82.8%).

**Conclusions/Significance:**

The OSP methodology may provide a valuable option for quality assessment of providers in places, or for health conditions, where other assessment tools are unworkable.

## Introduction

Assessing the quality of care provided by individual medical practitioners is critical to evaluating training, monitoring the scale up of programs delivering new treatments, and identifying possible risks to the health of the public. Provider quality assessments can identify where additional training, support, regulation or other improvements in health care are needed, or the effectiveness of current training programs. Information on provider practices is important both for population estimates of process quality, and for analysis of the determinants of quality.

Provider quality assessment is difficult in high-income countries despite well-established legal and regulatory frameworks and highly standardized reporting practices. In low-income countries the problems are made more difficult by uncertain training and regulatory standards and high variability among providers' levels of training or government levels of oversight. In rural areas these challenges are multiplied many fold. Assessing quality of treatment of pediatric illnesses is more difficult again. Nevertheless, in many low-income countries the more pervasive healthcare problems occur in rural settings and often are those illnesses that effect children. The need for interventions to improve or assure quality in these settings, across the range of providers delivery care, is of interest to public health practitioners.

The tools available for provider quality assessment in low- and middle-income countries fall into five basic categories, each with their own limitations: (1) Standardized Patients, (2) Clinical Vignettes, (3) Abstraction of Medical Records, (4) Medical Mannequins (5) and Direct Patient Observation.

Based upon an assessment of the strengths and weaknesses of the existing methods for provider assessment (described below), we felt that none of them was adequate to assess the quality of rural practitioners in the diagnosis and treatment of pediatric malaria in preparation for the expansion of community health workers training to diagnose and treat uncomplicated cases. To fill this gap, we developed a hybrid or synthesis method of provider assessment, combining components of direct observation, clinical vignettes, and medical mannequins which we have termed “Observed Simulated Patient” or OSP.

To test the validity of OSP to accurately measure the quality of medical outpatient care, we conducted a parallel scoring of medical providers in Myanmar, assessing the quality of their evaluation and treatment of pediatric malaria, first by direct observation of true patients and second by OSP.

### Traditional Quality Assessment Methods

#### A Standardized Patient

often called a patient actor, simulated patient, or mystery client, is “a person who has been carefully trained to take on the characteristics of a real patient in order to provide an opportunity for a student to learn or be evaluated firsthand” [1]. In standardized patient scenarios, the patient actor typically arrives unannounced to the practitioner and is responsible for completing an assessment checklist on the performance of the practitioner. In clinic scenarios, the practitioner is not aware that s/he is being evaluated by this particular patient [1], [2]. In medical education settings, students are usually aware of the evaluation being done.

Standardized patients are considered to be the gold standard for assessment of clinical skills and have been utilized for nearly 40 years in teaching medical curricula and today are incorporated into many medical education programs internationally [3], [4]. According to Peabody *et al*, the literature reviewed provides examples of how standardize patients can capture variation in clinical practice and reproducibly show how individual physician practices vary over time (12). The majority of the published studies were conducted in developed countries, but there are references to the use of this method in middle-income countries such as China and Ukraine [1], [5].

Credibility and cost are barriers to application in rural areas where non-local standardized patients may be easily identified and the combinations of training multiple actors, travel times, and transport make this an expensive assessment method. More significant are the challenges using standardize patients to assess practitioners in low- and middle- income countries are measuring diseases with fever or other obvious physiological presentation, conditions requiring invasive examinations, and pediatric illnesses where ethical considerations prevent patient recruitment.

#### Clinical Vignettes are hypothetical scenarios with questions or prompts for the chose course of action, given in stages to medical practitioners, with their responses noted for each stage before adding information [6]

Clinical vignettes are common components of medical education in many countries, and their application in both developed and developing countries has been shown in recent years to have high rates of internal validity [7]. An advantage of vignettes over other evaluation tools is that they allow cost effective measurement of relatively rare illnesses. For example, in Tanzania, vignettes have been used to assess provider diagnosis and treatment practices for tuberculosis as the infrequency of TB patient presentations in clinics makes direct client observation impractical [8].

Vignette scores are strongly correlated to inputs provided during consultation (rational history taking, physical examination, and health education) and the ability of the clinician to properly diagnose the presented illness [8]. The challenge is that growing evidence indicates that doctors do less with real patients than they say they would do in hypothetical scenario [8]–[10]. Vignettes are, then, a valid instrument to measure provider knowledge, but a much less effective tool for measuring the quality of provider practice. In situations where practitioners operate independently, with little regular oversight or interaction with others, this may be of particular concern; where multiple assessment methods are used the potential identification of differences between knowledge and practice may identify important perverse incentives. Alone, vignettes may miss important aspects of practice.

#### Abstraction of Medical Records is the most common way of evaluating physician practices, however application to outpatient care provided by rural practitioners has been limited, and data collection by trained professionals is expensive [11], [12]

There are questions about both the validity and feasibility of record abstractions as a tool for quality evaluation and tracking in non-hospital settings. In developing countries, medical record abstraction has been shown to be poorly correlated with standardized patient treatment – the gold standard for quality assessment [13]. Data from the US and from managers of clinical care programs in Africa and Asia indicate that individual practitioners are less likely to keep clinical records of any sort than providers operating in public facilities [14], [15]. In developing countries, informal practitioners and lower level providers such as midwives and nurses are also less likely to keep records than doctors [16]. Moreover, even where records are routinely kept, the method is dependent upon highly detailed information and clinical judgment being written down near the time of the patient encounter.

#### High fidelity Medical Mannequins are common tools for medical education in developed countries, both during formative training and in continuing medical education, notably in anesthesiology, surgery, obstetrics, emergency medicine, pediatrics (e.g., neonatal, infant, and child resuscitation), and critical care [24]

As with clinical vignettes, providers are given a scenario, and are instructed to diagnose and treat the mannequin as if it were a real person. The providers' treatment activities are assessed and scored by direct observation. Medical mannequins have principally been used for teaching and have seldom been used to assess medical competency practicing providers.

#### Direct Observation, or the observing or recording of a real-life patient, is a well-established method for performance-based assessment of clinical practitioners, and has been proven effective in developing countries for the assessment of outpatient care [17]

Direct Observation has been shown to provide an effective, and non-biased, tool for evaluating a range of practices [18]–[21]. The limitation of direct observation is the time and cost required, particularly in assessing rare illnesses, or in evaluating low-volume clinics, attributes of many rural facilities. In both situations, observers may have to wait days to observe a single provider-patient interaction meeting a study's inclusion criteria.

## Methods

This research was approved by the UCSF Committee on Human Research on October 26, 2010. A waiver of the requirement to obtain a signed consent form was given as “the research presents no more than minimal risk of harm to subjects and involves no procedures for which written consent is normally required outside of the research context.”

### Observed Simulated Patient

The present study combines and validates a new quality of care assessment method that combines elements of the above approaches to meet the needs of evaluating the quality of training and care for the scale up of treatment of pediatric malaria by lay community health workers in rural Myanmar. Our hybrid approach, called the Observed Simulated Patient (OSP), involves a trained actor playing the role of a ‘mother’, a life-size doll representing a 5-year old boy, a dramatized clinical vignette, and a trained observer. The provider is informed in advance of the role-playing, and told to conduct the assessment, make the diagnosis and provide treatment as he or she normally does, with the added instructions to verbally describe the examinations.

**Figure 1:**
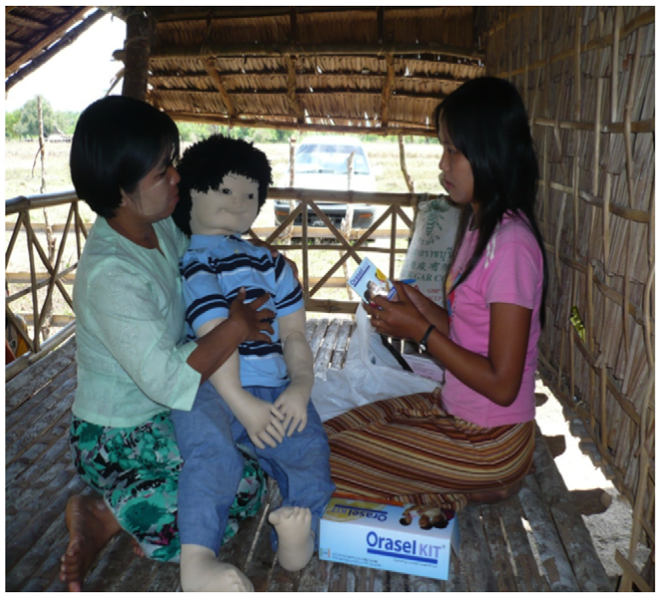
OSP testing of a rural provider.

The researcher, as the ‘mother’, presents herself, carrying the doll, and acts through a scenario describing the chief complaint of her child (the doll). At each stage, the mother provides a realistic but small amount of information, and the physician must assess the illness through inquiry and examination (Figure 1). Providers are not restricted from revisiting topics raised earlier in the examination if they wish to. If the provider announces that he would like to conduct a blood test, a simulated result of the test is provided: for example, if he chooses to conduct a rapid diagnostic test (RDT) for malaria, a test kit pre-marked with a result is provided. The provider then must interpret the RDT as he would during an ordinary exam and proscribe treatment.

**Figure 2:**
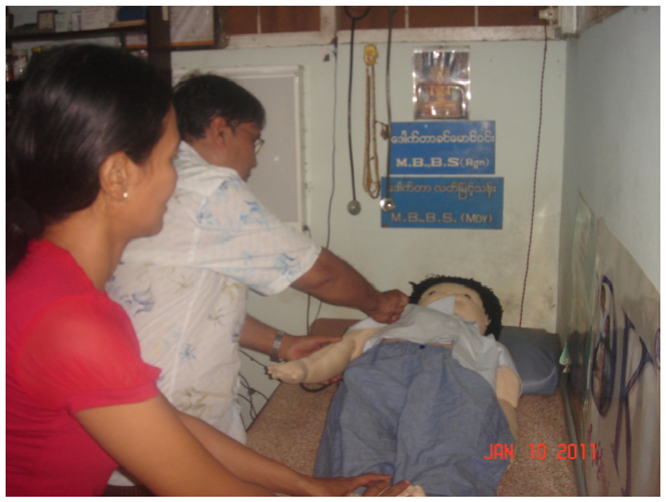
An urban provider examining the OSP mannequin.

The examination, diagnosis, and treatment are watched by a trained observer (Figure 2), and assessed using a scoring sheet divided into five sections reflecting the sets of tasks to be assessed (Table 1).

**Table 1:** Summary and Sub-unit evaluations scores for directly observed simulated patients: Reception and diagnosis.

Questionnaire Item	DO Patient	OSP Mannequin	Possible Score
	N = 20	N = 20	
**Unit 1: History Taking**			
Q 200 Duration of Fever	1	1	1
Q 201 Pattern of Fever	1	1	1
Q 202 Patient has diarrhoea	0.75	0.65	1
Q 203 Patient has runny nose	0.4	0.55	1
Q 204 Patient has cough	0.95	1	1
Total Score	4.1	4.2	5
**Unit 2: Severe signs of malaria**			
Q 205 examine eyes and nail beds	4	3.6	4
Q 206 examine Respiratory distress	0.75	0.1	1
Q 207 check ability to sit or walk without support	0	0.3	2
Q 208 examines whether unable to drink or vomits everything	1.2	1.7	2
Q 209 examine lethargic with convulsions, or been unconscious	0.6	1	2
Q 210 check passing of black water urine	0.2	1.1	2
Total Score	6.75	7.8	13
**Unit 3: Vital signs**			
Q 211 Taking Temperature	4	3.8	4
Q 212 Counting respiratory rate	0.4	0.4	4
Total Score	4.4	4.2	8
**Unit 4: Antimalaria drug history**			
Q 213 has taken malaria medicines in past 3 days	1.1	0.3	2
Q 214 bad response to malaria medicines before	0.45	0	3
Total Score	1.55	0.3	5
**Unit 5: Perform RDT kit**			
Q 215 administer RDT kit	5	5	5
Q 216 Correctly interprets result of RDT kit	20	19	20
Q 217 voluntarily inform result of RDT kit	5	5	5
Total Score	30	29	30
**Grand Total Section 1**	46.8	45.5	61

This study assessed the validity of OSP against direct observation in outpatient treatment settings in rural Myanmar. The study compared the two modes of measuring provider practices by scoring multiple aspects of examination, diagnosis, treatment, and counseling. In addition to validity, both methods of evaluation were assessed for suitability of evaluating infrequently performed tasks in difficult-to-reach settings. The study also measured, but was not powered to analyze, the ability of providers to reach the correct diagnosis with real and the OSP.

All practitioners were long-standing members of the Sun Quality Health network operated by PSI/Myanmar. Because of their affiliation with the network, all providers had been trained in management of pediatric malaria and were regularly supplied with rapid-diagnostic test kits (RDTs) and four different formulas of Coartem brand artemesinin-based combination therapy (ACT) for treatment of *p. falciparum* malaria in patients of all ages. All providers were also supplied with both chorloraquine and primaquine-chloraquine combined tablets for the treatment of *p. vivax* malaria. The RDTs used by the providers identify and distinguish *p. vivax* and *p. falciparum*.

Members of the Sun Quality Health network are private practitioners, recruited into a subsidized network with the intent of expanding access to affordable care. Providers are not paid but receive free training as part of membership, and have access to a limited number of subsidized medicines that they then sell on at below market prices to their patients. There is some indication that affiliation with Sun Quality Health enhances the reputation of the providers, and providers report an increase in client volume due to membership.

The providers in this study were selected because they had reported treatment of pediatric malaria in the prior year. Upon selection, providers were asked if they would agree first to have an observer score a real-life interaction with a suspected pediatric malaria patient, and second, to be assessed by the OSP method. A total of 37 providers agreed to participate, however the researchers were only able to observe 20 treating pediatric fever patients of similar age and presentation as the OSP tool. Only after a real patient had been observed did the researchers return to perform an OSP assessment, between one and five days later.

The tasks and descriptions used in the OSP scoring sheet were derived from WHO standards for appropriate diagnosis and treatment of malaria and the Myanmar Ministry of Health standards for care [22], [23]. The tasks covered history taking, general physical examination, assessment of vital signs, anti-malarial drug history collection, use and interpretation of RDT, and prescription of age and weight appropriate treatment. The scoring of each activity was developed by the researchers and weighed according to its clinical significance. Activity descriptions and weights were then adjusted following review by a panel of malaria experts at the Myanmar Institute of Tropical Medicine. The scoring sheet was developed in English, translated into Myanmar language, and back translated to English before being fielded. The same scoring sheet was used for the assessment of the simulated and actual malaria patients.

### Data Collection

Data on quality of care for children presenting with fever, and for the OSP presenting with fever, were collected from 20 private independent medical practitioners in Mon and Kayin States, Myanmar (two largely rural regions with endemic malaria) between December 26, 2010 and January 12, 2011.

Providers were contacted in person and told of the study, and then asked to telephone the research team staying nearby when a child with fever presented, before beginning the examination. A single team consisting of a senior medical educator and a trained researcher conducted all 40 assessments. The senior educator observed both real patients and the OSP interaction.

Data from observations were collected using paper records filled in by the observer, and all records were entered directly into SPSS 15.0 upon the team's return to Yangon. Time spent observing each encounter was also recorded. The study protocol allowed the observer to intervene after the consultation was completed if she felt a patient was being sent away without having been tested for a clinically mandated illness, or having received inappropriate or lacking care. In the event, this did not occur.

### Data Analysis and Interpretation

The evaluation of OSP in terms of its potential as a hybrid quality assessment tool and its reliability rests on three aspects of our study: a) its development following national and international guidelines with input of practitioners in the field on the relevance of the scenario, component items, and weights, b) the overall score among highly trained, knowledgeable, and experienced practitioners, and c) the agreement between OSP and real life patients. For the latter, we used the kappa statistic to compare the quality scores between directly observed patient care and the OSP assessment, gauging the level of agreement between the two measures for the same provider for the overall score and each of the five sub-components (history, examination, vital signs, drug history, and test performed). We used the Z test to assess the role of chance for the calculated kappa while correcting for the number of items using standard techniques in Stata software [24]. The size of the kappa reflects the level of agreement with the p-value assessing the likelihood of chance agreement.

Only provider responses that were spontaneous were included. As the OSP evaluation progressed, additional information was given to the providers to allow them to assess the specific features of malaria of interest. Provider questions that were un-related to malaria were not scored.

Only three of the 20 observed real patients were diagnosed to have malaria and treated. For this reason, the analysis compares assessment up to the point of diagnosis. Separately, we evaluated the performance of all 20 providers at providing appropriate treatment for malaria based upon the OSP scenario presented.

## Results

The providers, nearly universally, performed well at patient history taking and both using and reading the RDT kits of the OSPs. In both areas, the average observed patient score was more than 80% of the potential score. Providers did worse at asking about or identifying signs of sever malaria, and at taking patient vital signs. Providers most frequently omitted history taking specific to antimalarial drugs.

**Figure 3:**
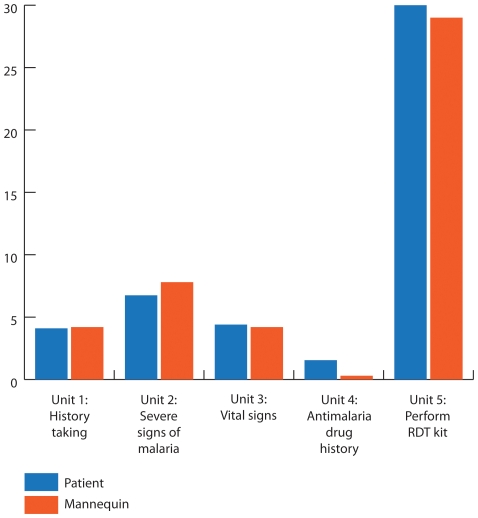
Average Provider scores when diagnosing patients and OSP mannequins. Twenty providers were scored on their performance diagnosing and treating malaria, first by direct observation with real patients; subsequently using the OSP methodology. Figure 3 shows the providers scored for each of the five diagnosis modules. The weights given to each module were determined through consultation with experts in malaria treatment as described in the text. Possible scores were: Unit 1: History Taking (5); Unit 2: Identify severe signs of malaria (13); Unit 3: Vital Signs (8); Unit 4: Antimalarial drug history (5); Unit 5: Perform Rapid Diagnostic Kit test (30).

As shown in Figure 3, there was little difference between average scores for true patients and OSP patients in all areas except antimalarial drug history taking. Only one measure, antimalarial drug history taking, differed by t-test between observed patients and the OSP.

Sub analysis of response areas shows similar rates of correlation between true patient and OSP scores (Table 1). By the kappa statistic and Z-test, there was statistically significant moderate agreement for the OSP tool overall (Table 2). By sub-components, there was significant perfect agreement in the critical area of RDT application and reading, significant moderate agreement in taking vital signs, borderline significant fair agreement in the general examination, and non-significant less agreement in the overall and in the drug taking histories.

**Table 2:** Difference in performance when caring for directly observed patients and observed simulated patients.

Components of care	R Pt (n = 20)	S Pt (n = 20)	t value	Significant level			
History taking	4.1	4.2	−0.384	NS			
General examination	6.75	7.8	−1.961	NS			
Taking vital signs	4.4	4.2	0.567	NS			
Asking anti-malarial drug history	1.55	0.3	2.877	*			
Perform rapid diagnostic tests	30	29	1	NS			
Total	46.8	45.5	1.033	NS			
* p<0.05							
		Expected					
**Indicators**	**Agreement**	Agreement	Kappa	Std. Err.	Z	Prob>Z	
History taking	91.88%	92.75%	−0.1207	0.2192	−0.55	0.709	Less agreement
General examination	94.59%	92.77%	0.2514	0.1942	1.29	0.0977	Fair agreement
Taking vital signs	95.82%	92.96%	0.4058	0.2193	1.85	0.0321	Moderate agreement
Anti-malarial drug history	82.8%	83.4%	−0.0377	0.0808	−0.47	0.6797	Less agreement
Perform rapid diagnostic tests	97.62%	97.6%	0	0	0	0.0	perfect agreement
Total	70.0%	50.0%	0.4	0.2191	1.83	0.0339	Moderate agreement

Among the 20 true patients only three had positive RDT tests and were treated for malaria. For this reason, we have not compared the treatment scores between real and OSP patients. The average scores of the providers with OSP patients on all clinical aspects of treatment were high, 30.25 out of a possible 32 (Table 3). As was true for the history-taking sub-module, providers scored poorly on prevention counseling of OSP patients, averaging 3.75 out of a possible 7.

**Table 3:** Summary and Sub-unit evaluations scores directly observed patients and observed simulated patients: Treatment and referral for malaria positive patients.

	DO Patient	OSP Mannequin	Possible Score
	N = 3	N = 20	
**Unit 6: Referral**			
Q 218 Refer to higher health facility	1	1	1
Total Score	1	1	1
**Unit 7: Weighing the patient**			
Q 219 Provider weighs patient	5	4.75	5
Q 220 Prescribing correct type of Coartem (Coartem 2)	5	4.75	5
Total Score	10	9.5	10
**Unit 8: Instruction to take Coartem**			
Q 221 Correctly advises when and how to give Coartem	4	3.8	4
Q 222 Advises for trouble taking solid pills and how to administer	4	3.8	4
Q 223 Provider says how long full course is (3 days)	4	3.8	4
Q 224 Provider emphasizes importance of taken ALL pills	5	4.75	5
Total Score	17	16.15	17
**Unit 9: Remind for follow-up**			
Q 225 Provider tells patient to bring child for F/up if the child doesn't get better or get worse	2.67	3.6	4
Total Score	2.67	3.6	4
**Unit 10: HE and counseling**			
Q 300 Advises on importance of early health seeking behavior with trained health provider	1	1.35	3
Q 301 Advises on importance of insecticide treated nets for prevention of malaria	2.67	2.4	4
Total Score	3.67	3.75	7
**Grand Total Section 2**	34.33	34	39

## Discussion

Our study suggests the high potential a new hybrid quality assessment tool when applied to the treatment of pediatric malaria in Myanmar. We found significant agreement in quality assessment scores among private providers when measuring their performance using direct observation of provider patient interaction and using Observed Simulated Patients. All areas of assessment showed agreement between OSP and direct observation above 90% except for history taking related to past experience with malaria medicines. In this area, providers did not ask questions of the OSP to the same degree that they questioned real patients (agreement 82.8%).

Of note, interpretation of the kappa statistic (achieving the conventional level of “moderate agreement” in our study) needs to take into account the very high level of overall correct responses among the trained practitioners. The overall quality of care provided by doctors in the study was found to be moderately high, averaging 79 out of a total possible score of 100, indicating broadly appropriate practice in diagnosis and treatment for the simulated patient. Providers lost points on history taking, but all providers scored well on use and interpretation of diagnostic test kits, and prescription of appropriate anti-malarial medicine. The kappa statistic can appear low when overall agreement is actually high because in the sample there is a high chance of getting correct responses, similar to how positive and negative predictive values of tests are affected by the prevalence of disease in the sample. This is a general limitation of the kappa statistic [25]. For the present study, we emphasize that the agreement is greater than predicted by chance in a context of high likelihood of a correct answer.

A budget-driven limitation of this study is the small sample size, and in particular the small number of confirmed malaria cases in the observed patient sample. Patients may have selected providers based on skills, and so it is possible that the 20 providers included in this study were more qualified than the 17 providers who did not report patients. We have also only compared the validity of OSP for one disease, in one setting. Caution must therefore be taken in making any extrapolations about the ability of OSP to provide accurate assessments of provider practices in the management of other health issues, or in countries where the norms of provider-patient interaction may be quite different from those in Myanmar and have correspondingly different responses to this methodology. The method of provider identifying febrile patients presenting may have introduced bias in which patients were included in the study. Providers in the study were scored on their ex-post treatment quality: in other words, they were not scored on treatment practices that were correct, but not related to malaria even where such examination might have allowed the providers to rule out non-malaria illnesses. This is a common gap in quality measurement, but remains unaddressed in this study.

In any assessment where the provider knows that he/she is being observed there is the possibility of a Hawthorne effect [26]. We have highlighted the risks of this in OSP as well as other quality assessment methods in Table 4. In this study, both methods evaluated were subject to the same problem and so we cannot know if the provider would have behaved in the same way had he/she was being observed. The providers' behavior may have been influenced by their ongoing relationship to PSI and Sun Quality Health. Providers receive benefits from membership in the Sun Quality Health network and may have provided better-than-normal care while under observation for this reason. Although this would effect both the Directly Observed patient and the OSP, there may have been differential impact on the first and second assessment. All providers in this study first were evaluated by DO, and later by OSP. A future study could alternate the order of DO and OSP assessments to determine this. We believe OSP to be cost-efficient compared to other methods based on the short time collecting data at each clinic and the continued use of a small number of trained researchers. Nevertheless, no comparison of the costs of conducting different quality assessment methods has been conducted leaving this issue, important for field implementation, unaddressed in this study.

**Table 4:** Comparison of Observed Simulated Patients and Existing Quality Measurement Tools.

Quality Measurement Tool	Measures Knowledge	Measures Practice	Accounts for Case-Mix	Accounts for patient-mix	Hawthorne effects?	Structural limitations
*Vignettes*	**Yes**	No	**Yes**	**Yes**	N/A: by design vignettes measure the maximum a provider can do	none
*Clinical Observation*	**Yes**	**Yes**	No	No	Yes: large Hawthorne effects to begin with; decline with the time spent observing	(a) Hard to observe as “serious” illnesses as most are rare; (b)observer never knows true patient diagnosis
*Chart Abstraction*	**Yes**	**Yes**	No	No	No	Infeasible for private sector: providers don't keep patient charts
*Standardized Patients*	**Yes**	**Yes**	**Yes**	**Yes**	No	Limited to: (a) non-infectious diseases; (b) adults only; (c) diseases without obvious physiological symptoms that cannot be mimicked; (d) conditions that don't require invasive exams
*Observed Simulated Patients*	**Yes**	**Yes**	**Yes**	**Yes**	unknown	none

Despite these limitations the close degree of quality score correlation between OSP measures and observed patient measures suggests that this methodology may provide a valuable option for quality assessment of providers in places, or for health conditions, where other assessment tools are impossible or impractical. While a variety of quality assessment methods may be used in urban areas, measuring the quality of care provided by rural providers is challenging for a range of reasons. For example, the language, ethnicity, likelihood of personal provider-patient knowledge, and the ethical and practical barriers associated with pediatric illnesses make introducing external mystery clients impossible. Small patient volumes and long distances between providers make direct observation costly. And poor or incomplete paper record keeping in many small clinics obviates record abstraction. OSP has the potential to provide a solution to these challenges.

The source of care in developing countries includes a wide range of providers, from qualified doctors to informal providers. It is desirable, therefore, for an assessment tool to be used to assess the presentation of complicated illnesses in a range of settings. Although not part of this evaluation, we feel that the applicability of assessment tools across a range of provider types deserves study going forward.

The value of a quality assessment tool can be conceptualized as (a) the extent to which they are able to provide information on a broad set of illnesses; (b) the extent to which they are able to provide estimates that account for confounders and; (c) the extent to which they measure knowledge versus practice. As described above, a number of current tools are limited in the conditions they are able to assess, or the patient populations they can mimic (Table 4). In this context, the results from this study lead us to believe that OSP offers an advantage on existing quality assessment tools in some instances, and merit a larger pilot of the use of OSP to assess the quality of management of pediatric malaria by rural medical practitioners is merited. The use of this methodology has the potential to provide an accurate and affordable solution to the challenges of rural outpatient quality assessment.

## References

[1] Wallace P (1997). Following the threads of an innovation: the history of standardized patients in medical education.. CADUCEUS-SPRINGFIELD-.

[2] Shah R, Edgar D, Evans BJ (2007). Measuring clinical practice.. Ophthalmic Physiol Opt.

[3] Badger LW, deGruy F, Hartman J, Plant MA, Leeper J (1995). Stability of standardized patients' performance in a study of clinical decision making.. Fam Med.

[4] Pieters HM, Touw-Otten F, Melker RA (1994). Simulated patients in assessing consultation skills of trainees in general practice vocational training: a validity study.. Medical Education.

[5] Stillman PL, Wang Y, Ouyang Q, Zhang S, Yang Y (1997). Teaching and assessing clinical skills: a competency-based programme in China.. Medical education.

[6] Luck J, Peabody JW, Lewis BL (2006). An automated scoring algorithm for computerized clinical vignettes: evaluating physician performance against explicit quality criteria.. Int J Med Inform.

[7] Peabody JW, Tozija F, Munoz JA, Nordyke RJ, Luck J (2004). Using vignettes to compare the quality of clinical care variation in economically divergent countries.. Health Serv Res.

[8] Leonard KL, Masatu MC, Vialou A (2007). Getting doctors to do their best: the roles of ability and motivation in health care quality.. Journal of Human Resources.

[9] Das J, Hammer J, Leonard K (2008). The quality of medical advice in low-income countries.. J Econ Perspect.

[10] Rethans JJ, Sturmans F, Drop R, van der Vleuten C, Hobus P (1991). Does competence of general practitioners predict their performance? Comparison between examination setting and actual practice.. BMJ.

[11] Reisch LM, Fosse JS, Beverly K, Yu O, Barlow WE (2003). Training, quality assurance, and assessment of medical record abstraction in a multisite study.. Am J Epidemiol.

[12] Hong TT, Walker SM, McKenzie K (2009). The quality of injury data from hospital records in Vietnam.. HIM J.

[13] Peabody JW, Luck J, Glassman P, Dresselhaus TR, Lee M (2000). Comparison of vignettes, standardized patients, and chart abstraction: a prospective validation study of 3 methods for measuring quality.. JAMA.

[14] Thompson HC, Osborne CE (1976). Office records in the evaluation of quality of care.. Med Care.

[15] Charman N, Hovig D, Ahmed R (2010). personal communication from clinical care program managers working with private practitioners in developing countries..

[16] Shah NM, Brieger WR, Peters DH (2011). Can interventions improve health services from informal private providers in low and middle-income countries? A comprehensive review of the literature.. Health Policy Plan.

[17] Franco LM, Franco C, Kumwenda N, Nkhoma W (2002). Methods for assessing quality of provider performance in developing countries.. Int J Qual Health Care.

[18] Kogan JR, Holmboe ES, Hauer KE (2009). Tools for direct observation and assessment of clinical skills of medical trainees: a systematic review.. JAMA.

[19] Leonard KL, Masatu MC (2005). The use of direct clinician observation and vignettes for health services quality evaluation in developing countries.. Soc Sci Med.

[20] Onishi J, Gupta S, Peters DH (2011). Comparative analysis of exit interviews and direct clinical observations in pediatric ambulatory care services in Afghanistan.. Int J Qual Health Care.

[21] Schnelle JF, Ouslander JG, Simmons SF (2006). Direct observations of nursing home care quality: Does care change when observed?. J Am Med Dir Assoc.

[22] Ministry of Health M (2008).

[23] Organization WH (2009). Guidelines for diagnosis and management of malaria in Myanmar.

[24] Fleiss JL (1981). Statistical methods for rates and proportions.

[25] Viera AJ, Garrett JM (2005). Understanding interobserver agreement: the kappa statistic.. Fam Med.

[26] Leonard KL (2008). Is patient satisfaction sensitive to changes in the quality of care? An exploitation of the Hawthorne effect.. J Health Econ.

